# Lrp4 in hippocampal astrocytes serves as a negative feedback factor in seizures

**DOI:** 10.1186/s13578-020-00498-w

**Published:** 2020-11-23

**Authors:** Zheng Yu, Meiying Zhang, Bin Luo, Hongyang Jing, Yue Yu, Shunqi Wang, Shiwen Luo

**Affiliations:** 1 Center for Experimental Medicine, The First Affiliated Hospital of Nanchang University, Nanchang, 330006 Jiangxi China; 2 Institute of Life Science and School of Life Sciences, Nanchang University, Nanchang, 330006 Jiangxi China; 3 Nanchang University Hospital, Nanchang University, Nanchang, 330006 Jiangxi China; 4 Teensen Genesis School, Nanchang, 330006 Jiangxi China; 5 Jiangxi Key Laboratory of Molecular Diagnostics and Precision Medicine, 17 Yongwai Street, Donghuo Distinct, Nanchang, 330006 Jiangxi China

**Keywords:** Low-density lipoprotein receptor-related protein 4, Epilepsy, microRNA, Astrocytic NMDA receptor, The threshold of seizure

## Abstract

**Background:**

Epilepsy is characterized by the typical symptom of seizure, and anti-seizure medications are the main therapeutic method in clinical, but the effects of these therapy have not been satisfactory. To find a better treatment, it makes sense to further explore the regulatory mechanisms of seizures at genetic level. Lrp4 regionally expresses in mice hippocampus where is key to limbic epileptogenesis. It is well known that neurons release a high level of glutamate during seizures, and it has been reported that Lrp4 in astrocytes down-regulates glutamate released from neurons. However, it is still unclear whether there is a relationship between Lrp4 expression level and seizures, and whether Lrp4 plays a role in seizures.

**Results:**

We found that seizures induced by pilocarpine decreased Lrp4 expression level and increased miR-351-5p expression level in mice hippocampus. Glutamate reduced Lrp4 expression and enhanced miR-351-5p expression in cultured hippocampal astrocytes, and these effects can be partially attenuated by AP5. Furthermore, miR-351-5p inhibitor lessened the reduction of Lrp4 expression in glutamate treated hippocampal astrocytes. Local reduction of Lrp4 in hippocampus by sh *Lrp4* lentivirus injection in hippocampus increased the threshold of seizures in pilocarpine or pentylenetetrazol (PTZ) injected mice.

**Conclusions:**

These results indicated that high released glutamate induced by seizures down-regulated astrocytic Lrp4 through increasing miR-351-5p in hippocampal astrocytes via activating astrocytic NMDA receptor, and locally reduction of Lrp4 in hippocampus increased the threshold of seizures. Lrp4 in hippocampal astrocytes appears to serve as a negative feedback factor in seizures. This provides a new potential therapeutic target for seizures regulation.

## Background

Epilepsy is one of the most neurological diseases, which affects over 50 million people worldwide [1]. The definition of Epilepsy is a disorder of the brain characterized by an enduring predisposition to generate epileptic seizures. In the clinical, it is usually practically applied as having two unprovoked seizures > 24 h apart [2]. The main goal of treatment for epilepsy is to stop seizures, reduce morbidity and decrease the risk of premature mortality associated with continuing seizures [3]. Although over 25 anti-seizure medications are currently used in clinical, only about 66% patients respond to these drug [4]. In reality, more than half of those taking epilepsy medication were still having seizures [5]. So, it is important to explore new anti-seizure methods. To achieve this goal, it makes sense to explore the regulatory mechanisms of seizures at genetic level.

Lrp4 is a type I single transmembrane protein of the LDLR family with a large extracellular domain, which expresses widely in many organs of mice, such as muscles [6], brain [7, 8], bone [9–11], mammary and other skin appendage placodes [12], and kidney [13]. In muscle, Lrp4 mainly expresses in the neuromuscular junction (NMJ) where is the site for the transmission of action potential from nerve to the muscle. Lrp4 serves as a receptor for AGRIN and is critical for the formation and maintenance of the NMJ [14]. In brain, Lrp4 regionally expresses in hippocampal astrocytes, and astrocytic Lrp4 maintains glutamatergic transmission by controlling the release of ATP from astrocytes. Hippocampus is an area where is key importance for limbic epileptogenesis, and *GFAP-Lrp4*^−/−^ mice had a raised threshold to seizures [7, 8]. So, the question here is whether there is a relationship between Lrp4 expression and seizures, and whether Lrp4 plays a role in seizures.

Animal seizures model has been widely employed to evaluate the progression of seizures, and used to study how seizures regulate gene expression in brain [15, 16]. It has been reported that pilocarpine treated rodents provokes a rapid and prolonged seizure, and leads to several molecular and cellular changes in hippocampus [17, 18]. We found that high released glutamate in seizures induced by pilocarpine down-regulated astrocytic Lrp4 expression through increasing miR-351-5p expression level in hippocampal astrocytes via activating astrocytic NMDA receptor. Furthermore, the local reduction of Lrp4 induced by sh *Lrp4* lentivirus injection in mice hippocampus increased the threshold of seizures in pilocarpine or pentylenetetrazol (PTZ) injected mice.

## Results

### Seizures induced by pilocarpine decreased Lrp4 expression in hippocampus in vivo

**Fig. 1:**
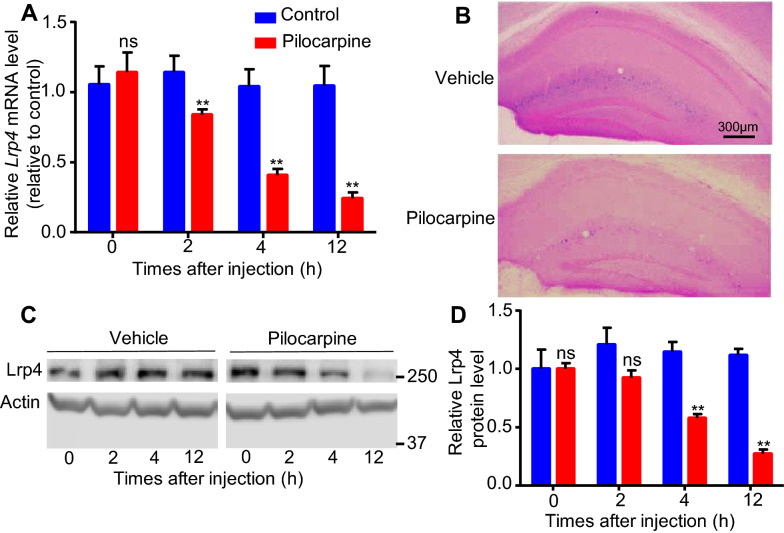
Seizures induced by pilocarpine decreased Lrp4 expression in mice hippocampus. **a** Relative mRNA level of *Lrp4* in the hippocampus of pilocarpine injected mice, which were collected at different time point (0 h, 2 h, 4 h, 12 h) after injection. Comparing to control mice (vehicle injection), *Lrp4* mRNA decreased after injection (2 h, 4 h, 12 h). **b** X-gal staining the brain slice of pilocarpine injected *Lrp4-lacZ*/+ mice. β-Gal activity decreased in both stratum lacunosum-moleculare layer (LMol) and molecules layer (Mol) of hippocampus. **c** Lrp4 protein level in the hippocampus of pilocarpine injected mice, which were collected at different time point (0 h, 2 h, 4 h, 12 h) after injection, Actin as an internal control. **d** Quantification of Lrp4 protein level in **c**, Lrp4 protein decreased after injection (4 h, 12 h). For each experiment, three separate experiments were performed in duplicate (**p < 0.01)

To find out whether seizures could influence Lrp4 expression in hippocampal astrocytes, epileptic seizures were induced in adult mouse by pilocarpine injection (300 mg/kg, i.p.) [19]. Under our conditions, discontinuous seizures began to develop at around 20 min after the injection and lasted up to 4 h. Pilocarpine injected mice and controls (saline injected) were sacrificed at different time 0 h, 2 h, 4 h, 12 h after injection (each time point, n = 3, per group), and total mRNAs of hippocampus were isolated for RT-qPCR. The results showed that seizures induced by pilocarpine significantly increased the relative level of *Bdnf* mRNA in hippocampus at each time point after injection, compared to control mice (injected with saline) (Additional file 1: Fig. S1A), which is in agreement with previous observations [20, 21]. This result indicated that pilocarpine induced seizures regulated gene expression in hippocampus. Intriguingly, compared to control mice, the relative level of *Lrp4* mRNA in hippocampus was significantly decreased in pilocarpine injected mice. After injection, relative *Lrp4* mRNA level began to decrease at 2 h and move on to 12 h with further reduction (Fig. 1a). To confirm this result, we used *Lrp4-lacZ*/+ heterozygote mice [7, 8], in which the *Lrp4* gene was replaced with a cassette encoding a β-galactosidase (β-gal) fusion protein on one chromosome, to proceed pilocarpine induced seizure experiment. 4 h after pilocarpine injection, mice were sacrificed and brain slices were stained by X-gal. The results showed that β-gal activity was significantly reduced in stratum lacunosum-moleculare layer (LMol) and molecules layer (Mol) of the hippocampus (most of them are astrocytes), compared to control mice (Fig. 1b). This result also indicated that seizures induced by pilocarpine significantly decreased *Lrp4* mRNA level in hippocampal astrocytes. Furthermore, after injected pilocarpine, total proteins of hippocampus were extracted at different times by RIPA and resolved by SDS-PAGE and subjected to Western blot analysis. The results showed that the relative level of Lrp4 protein in hippocampus were also significantly decreased in pilocarpine injected mice, but it began at 4 h, later than the time of the reduction of *Lrp4* mRNA level (Fig. 1c, d). These results indicated that seizures induced by pilocarpine significantly decreased Lrp4 expression level (both mRNA and protein) in hippocampus in vivo.

### Glutamate decreased Lrp4 expression of hippocampal astrocytes in vitro

**Fig. 2:**
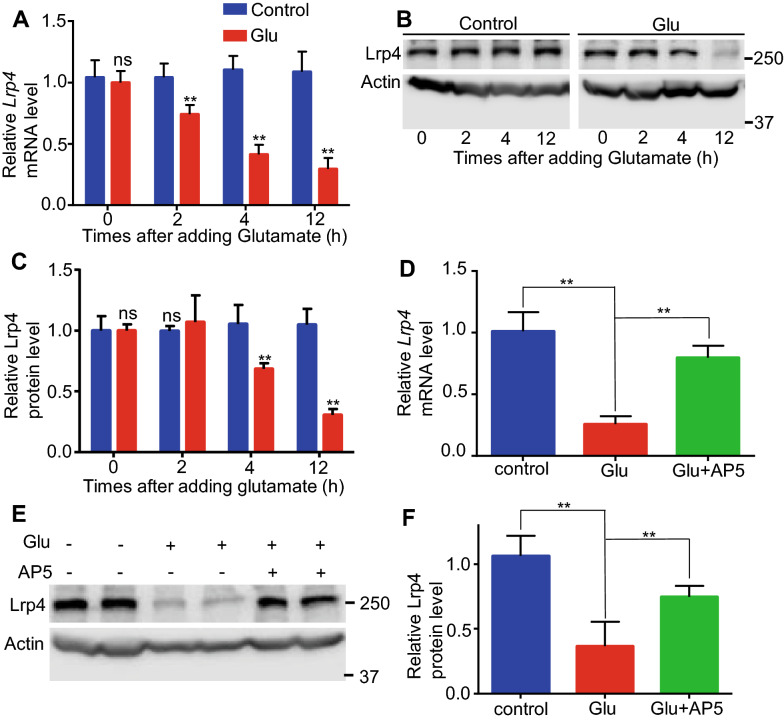
Glutamate decreased Lrp4 expression through activating NMDA receptor in cultured hippocampal astrocytes. **a** Relative mRNA of *Lrp4* in cultured hippocampal astrocytes after glutamate treatment, which were collected at different time point (0 h, 2 h, 4 h, 12 h) after treatment. *Lrp4* mRNA decreased after glutamate treatment (2 h, 4 h, 12 h). **b** Protein level of Lrp4 in cultured hippocampal astrocytes after glutamate treatment, which were collected at different time point (0 h, 2 h, 4 h, 12 h) after treatment, Actin as an internal control. Lrp4 protein decreased after glutamate treatment (4 h, 12 h). **c** Quantification of Lrp4 protein level in **b**. **d** Relative mRNA level of *Lrp4* in primary cultured astrocytes which were treated with glutamate (0.5 mM) only or glutamate (0.5 mM) plus AP5 (50 mM). After 12 h, *Lrp4* mRNA decreased about 75% after glutamate treatment, but decreased about 20% after glutamate plus AP5 treatment. **e** Lrp4 protein level in cultured astrocytes which were treated with glutamate (0.5 mM) only or glutamate (0.5 mM) plus AP5 (50 mM). Actin as an internal control in Western blotting. **f** Quantification of Lrp4 protein level in **e**, Lrp4 protein decreased about 70% after glutamate treatment, but decreased about 25% after glutamate plus AP5 treatment. For each experiment, three separate experiments were performed in duplicate (**p < 0.01)

To better investigate the mechanism of previous finding, we tried to find a way to mimic pilocarpine induced seizures in vitro. Firstly, primary cultured hippocampal astrocytes were used in our study, because most of Lrp4 expresses in astrocytes [7, 8]. Then, glutamate (0.5 mM) treatment in cultured hippocampal astrocytes was used, because it has been reported that pilocarpine induces an elevation in glutamate levels in the hippocampus, following the appearance of pilocarpine induced seizures [22], and the increase in the glutamate efflux was found in hippocampal synaptosomes [23], the increase of glutamate release influences gene expression level in astrocytes [24]. 4 h after glutamate treatment, total mRNAs of cultured hippocampal astrocytes were isolated for RT-qPCR. The result showed that the relative level of *Bdnf* mRNA in glutamate treated astrocytes significantly increased, compared to control (vehicle treatment) (Additional file 1: Fig S1B). The result was in agreement with previous report [24] and our previous data in vivo (Fig. 1a). This indicated that glutamate can be used to treat cultured hippocampal astrocytes to mimic the process that pilocarpine induced seizures influences gene expression level in hippocampal astrocytes in vivo. To detect whether *Lrp4* mRNA level in cultured hippocampal astrocytes could be changed by glutamate, mRNA was collected at different times after glutamate treatment. The result showed that the relative mRNA level of *Lrp4* began to decrease about 30% at 2 h, and about 60% at 12 h (Fig. 2a). At the protein level, the relative level of Lrp4 protein in hippocampal astrocytes has no significantly changes at 2 h after glutamate treatment, but began to decrease 30% at 4 h and then decreased 50% at 12 h (Fig. 2b, c). These results indicated that glutamate decreased the Lrp4 expression level in cultured hippocampal astrocytes.

### Glutamate decreased Lrp4 expression in cultured hippocampal astrocytes through activating astrocytic NMDA receptor

To investigate the mechanism of the reduction of Lrp4 expression in glutamate treated hippocampal astrocytes, cultured hippocampal astrocytes were treated with glutamate (0.5 mM) with or without AP5 (50 mM) simultaneously. After 12 h, compared to control, the relative level of *Lrp4* mRNA decreased about 70% in astrocytes which were treated by glutamate without AP5, but only decreased about 20% in astrocytes which were treated by glutamate with AP5 (Fig. 2d). We also detected the change of Lrp4 protein level in this assay. After 12 h, the reduction of Lrp4 protein in glutamate with AP5 treated hippocampal astrocytes was also less than that in glutamate without AP5 treated hippocampal astrocytes (Fig. 2e, f). These results showed that NMDA receptor antagonist AP5 can partially attenuated the reduction of *Lrp4* mRNA and Lrp4 protein expression level in glutamate treated astrocytes. This indicated that glutamate decreased Lrp4 expression in cultured hippocampal astrocytes through activating astrocytic NMDA receptor.

### Seizures induced by pilocarpine increased miR-351-5p expression in mice hippocampus in vivo

**Fig. 3:**
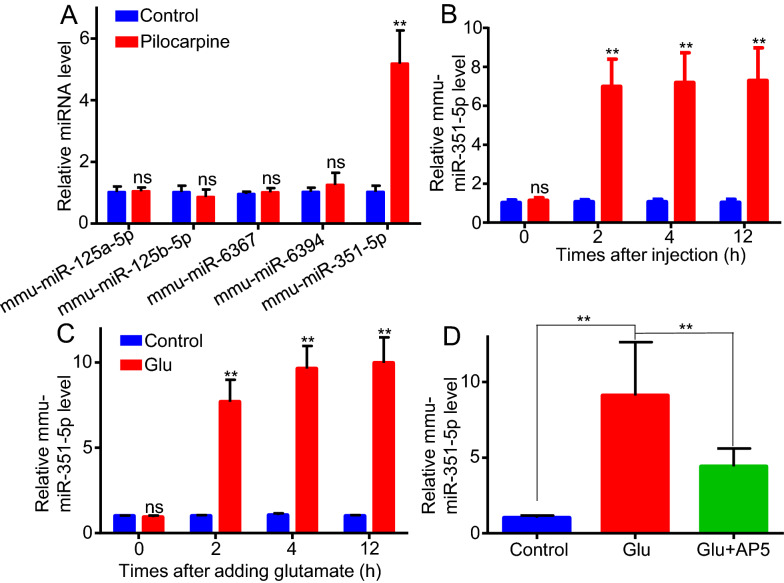
Seizures increased miR-351-5p in vivo and glutamate increased miR-351-5p in vitro through activating NMDA receptor. **a** Relative level of microRNA in hippocampus, injecting pilocarpine into mice. 4 h after injection, the relative level of mmu-miR-351-5p in hippocampus was increased. Two separate experiments were performed in triplicate (**p < 0.01). **b** Time course of the mmu-miR-351-5p relative level in hippocampus after injection, mmu-miR-351-5p increased at each time point (2 h, 4 h, 12 h) after injection. **c** Time course of the mmu-miR-351-5p relative level in hippocampus after glutamate treatment. mmu-miR-351-5p increased at each time point (2 h, 4 h, 12 h) after treating. **d** Relative level of mmu-miR-351-5p expression in hippocampal astrocytes which were treated with glutamate (0.5 mM) only or glutamate (0.5 mM) plus AP5 (50 mM). After 4 h treating, the elevation of mmu-miR-351-5p induced by glutamate plus AP5 treating is significantly lower than that induced by glutamate treating. For each experiment, three separate experiments were performed in duplicate (**p < 0.01)

MicroRNAs play important gene-regulatory roles in animals and direct their posttranscriptional repression by pairing to the mRNAs of protein-coding genes [25]. TargetScan website is the most widely used website for predicting microRNAs to regulate protein expression [26]. Through this website’s query, we found top 5 predict microRNA which target to mouse *Lrp4* gene: mmu-miR-125a-5p, mmu-miR-125b-5p, mmu-miR-351-5p, mmu-miR-6367, mmu-miR-6394. Then we detected whether seizures induced by pilocarpine could regulate the expression of these 5 microRNAs in mice hippocampus. After 4 h injection of pilocarpine or saline (vehicle control), mice were sacrificed, and total RNAs of hippocampus were isolated and stem-loop micro-RNA RT-qPCR assay was used to analyze microRNA. Quantitation cycle (Cq) values of microRNA were normalized to U6 small nuclear RNA, which was used as an internal control. The result showed that pilocarpine only significantly increased the relative level of mmu-miR-351-5p in hippocampus, but not those of mmu-miR-125a-5p, mmu-miR-125b-5p, mmu-miR-351-5p, and mmu-miR-6367 (Fig. 3a). These results were consistent with previous report [27]. Then we detected the increase of mmu-miR-351-5p level in hippocampus induced by seizures at different times. The result showed that compared to control, mmu-miR-351-5p expression began to increase around 6 times at 2 h after pilocarpine injection, and the elevation persisted for more than 12 h (Fig. 3b), which were simultaneous with the reduction of Lrp4 expression in hippocampus. These results indicated that seizures induced by pilocarpine increased mmu-miR-351-5p level in mice hippocampus.

### Glutamate increased mmu-miR-351-5p level in cultured hippocampal astrocytes through activating astrocytic NMDA receptor in vitro

Again, we wanted to detect whether glutamate can increase mmu-miR-351-5p expression in cultured hippocampal astrocytes. After treated with glutamate (0.5 mM), mmu-miR-351-5p level in cultured hippocampal astrocytes was increased about 7 times at 2 h after glutamate treatment, and the elevation persisted for more than 12 h (Fig. 3c), which was consistent with previous data in vivo. Furthermore, cultured hippocampal astrocytes were treated with glutamate (0.5 mM) without or with AP5 (50 mM) at the same time. After 12 h, total RNAs of astrocytes were isolated for micro RNA RT-qPCR. The results showed that the elevation of mmu-miR-351-5p level in glutamate with AP5 treated astrocytes is significant lower than that in glutamate without AP5 treated astrocytes (Fig. 3d). These results indicated that glutamate increased the expression of mmu-miR-351-5p in cultured hippocampal astrocytes through activating astrocytic NMDA receptor.

### miR-351-5p mimics decreased Lrp4 expression in cultured hippocampal astrocytes in vitro

**Fig. 4:**
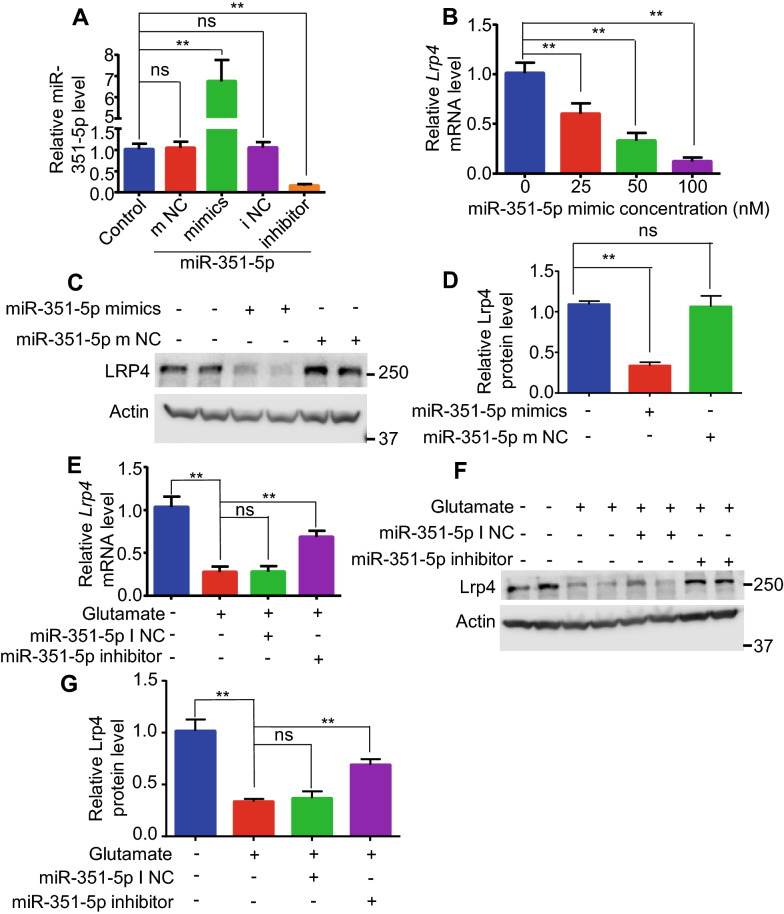
miR-351-5p inhibitor attenuated the reduction of Lrp4 expression by glutamate treating in cultured hippocampal astrocytes. **a** Relative level of mmu-miR-351-5p in hippocampal astrocytes which were transfected respectively by miR-351-5p mimics, miR-351-5p m NC (mimics negative control), miR-351-5p inhibitor, miR-351-5p I NC (inhibitors negative control). miR-351-5p mimics enhanced the miR-351-5p level and miR-351-5p inhibitors weakened the miR-351-5p level. **b** Relative level of *Lrp4* mRNA in primary cultured hippocampal astrocytes which were transfected with miR-351-5p mimics at different concentration (0 nM, 25 nM, 50 nM, 100 nM). *Lrp4* mRNA level decreased at the test concentration of the mimics (25 nM, 50 nM, 100 nM). **c** Lrp4 protein level in cultured astrocytes after transfecting miR-351-5p mimics by Western blotting. Action as an internal control. **d** Quantification of Lrp4 protein level from panel C, miR-351-5p mimics cut down Lrp4 protein expression. **e** mRNA level of *Lrp4* in astrocytes after glutamate treatment only or glutamate treatment plus miR-351-5p inhibitors transfection. mRNA level of *Lrp4* in astrocytes decreased about 70% by glutamate treating, and decreased about 25% by glutamate plus miR-351-5p inhibitors treating. **f** Lrp4 protein level in cultured astrocytes after glutamate treatment only or glutamate treatment plus miR-351-5p inhibitors transfection, Actin as an internal control. **g** Quantification of Lrp4 protein level from **f**, Lrp4 protein in astrocytes decreased about 70% by glutamate treating and decreased about 25% by glutamate plus miR-351-5p inhibitors treating. For each experiment, three separate experiments were performed in duplicate (**p < 0.01)

Previous data showed that seizures or glutamate increased miR-351-5p and reduced Lrp4 concurrently in hippocampal astrocytes in vivo or in vitro. The question is whether the reduction of Lrp4 level is due to the increasing of miR-351-5p level in hippocampal astrocytes. To answer this question, cultured hippocampal astrocytes were transfected respectively with miR-351-5p mimics or miR-351-5p m NC (control mimics) (50 nM). After 24 h, the miR-351-5p level significantly increased in miR-351-5p mimics transfected astrocytes compared to control (not transfected astrocytes), but no change in miR-351-5p m NC transfected astrocytes. The results showed that miR-351-5p level was successfully increased in miR-351-5p mimics transfected hippocampal astrocytes (Fig. 4a). Then different concentration of miR-351-5p mimics (0 nM, 25 nM, 50 nM, 100 nM) were transfected into cultured hippocampal astrocytes. After 24 h, the relative level of *Lrp4* mRNA were measured by qPCR. The result showed that the relative level of *Lrp4* mRNA was decreased at each concentration level (25 nM, 50 nM, 100 nM) (Fig. 4b). Because the reduction of relative *Lrp4* mRNA had reached about 70% in 50 nM miR-351-5p mimics treatment astrocytes, we decided to transfect 50 nM miR-351-5p mimics or miR-351-5p m NC into cultured hippocampal astrocytes to do next experiments. After transfected by miR-351-5p mimics, the relative Lrp4 protein level in cultured hippocampal astrocytes was significantly decreased compared to control, but no change in miR-351-5p m NC transfected astrocytes (Fig. 4c, d). These results indicated that Lrp4 expression level in cultured hippocampal astrocytes could be suppressed by miR-351-5p mimics transfection.

### miR-351 inhibitors attenuated Lrp4 expression reduction induced by glutamate in cultured hippocampal astrocytes

To further confirm that the reduction of Lrp4 expression level in astrocytes is due to the increasing of miR-351-5p, cultured hippocampal astrocytes were transfected with miR-351-5p inhibitors or miR-351-5p I NC (control inhibitors) (50 nM respectively). After 24 h, the miR-351-5p level significantly decreased in miR-351-5p inhibitors transfected astrocytes compared to control (not transfected astrocytes), but there was no change in miR-351-5p I NC transfected astrocytes (Fig. 4a). The result indicated that miR-351-5p expression in hippocampal astrocytes can be successfully suppressed by miR-351-5p inhibitors. Then we transfected miR-351-5p inhibitors or miR-351-5p I NC (50 nM respectively) into cultured hippocampal astrocytes. After 24 h, astrocytes were treated with glutamate (50 mM) for 4 h. The mRNA or protein were collected and subjected to qPCR or western blot. The results showed that compared to control astrocytes, both the relative level of *Lrp4* mRNA and Lrp4 protein decreased about 75% in the glutamate treated astrocytes and glutamate treated with miR-351-5p miR-351-5p I NC transfected astrocytes, but only decreased about 25% in glutamate treated with miR-351-5p inhibitors transfected astrocytes (Fig. 4e–g). These results showed that miR-351-5p inhibitors attenuated the reduction of Lrp4 expression level in glutamate treated hippocampal astrocytes. Combine with previous data, it was proven that the reduction of Lrp4 expression level in glutamate treated hippocampal astrocytes was caused by the increase of mmu-miR-351-5p.

### Low expression of Lrp4 in the hippocampus of mice trans-infected by sh *Lrp4* lentivirus enhanced the threshold of seizure

**Fig. 5:**
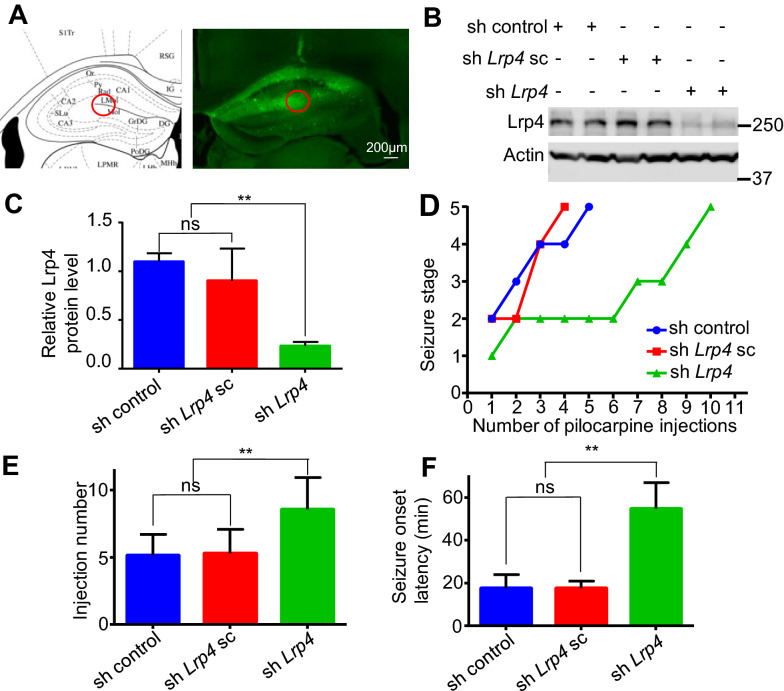
The reduction of Lrp4 expression in hippocampus increased the threshold of seizures. **a** Schematic diagram of the site of stereotactic injection of lentivirus. The expression of GFP showed that virus was precisely injected into lacunosum-moleculare layer (LMol) and molecules layer (Mol) of the hippocampus in mice. **b** Lrp4 protein level of the hippocampus in the mice, which were trans-infected by sh control lentivirus (sh pll3.7 vector injection), sh *Lrp4* scramble lentivirus, sh *Lrp4* lentivirus, Actin as an internal control in Western blotting. **c** Quantification of Lrp4 protein level in **b**. Lrp4 protein expression decreased about 70% in sh *Lrp4* lentivirus trans-infected mice. Three separate experiments were performed in duplicate (**p < 0.01). **d** Representative time courses of seizure development by repeated pilocarpine injection. Different virus trans-infected mice were subjected to pilocarpine injection every 30 min and seizure stages were scored at the same time. **e** Increased number of pilocarpine injections were needed to reach stage 5 seizure for sh *Lrp4* lentivirus trans-infected mice (10 injections), comparing with sh control lentivirus (5 injections) and sh *Lrp4* scramble lentivirus (5 injections) trans-infected mice. (n = 10 mice for each group; **p < 0.01). **f** Increased latency of sh *Lrp4* lentivirus trans-infected mice to generalized convulsive seizure in response to PTZ. (n = 10 mice for each group; **p < 0.01)

Could the reduction of Lrp4 expression level suppress seizures? We stereotactic injected shRNA lentivirus (sh control, sh *Lrp4* scramble, sh *Lrp4*) into lacunosum-moleculare layer (LMol) and molecules layer (Mol) regions of mice hippocampus (Fig. 5a). The mice were allowed to recover for 14 days. Total proteins of hippocampus were extracted by RIPA and resolved by SDS-PAGE and subjected to Western blot analysis. The result showed that sh *Lrp4* lentivirus trans-infection significantly decreased the relative Lrp4 protein level in mice hippocampus, compared to control (sh control lentivirus injection), but not sh *Lrp4* scramble lentivirus trans-infection. The results indicated the effectiveness of sh *Lrp4* (decreasing Lrp4 expression level) (Fig. 5b, c). 2 weeks after lentivirus injection for recovery, we injected mice with pilocarpine (200 mg/kg, i.p.), and followed additional injections of pilocarpine (100 mg/kg) every 30 min. Most control mice (sh control lentivirus trans-infected mice) and sh *Lrp4* scramble lentivirus trans-infected mice developed status epilepticus to score 5 (violent convulsions, falling over, death) after the fifth injection. In contrast, a majority of sh *Lrp4* lentivirus trans-infected mice did not develop status epilepticus to score 5 even after the ninth injection (Fig. 5d, e). These data indicated that sh *Lrp4* lentivirus trans-infected mice had a raised threshold to seizure due to reduction of Lrp4 in hippocampus. After injecting mice with PTZ, a GABAA receptor antagonist that induces seizure via different mechanisms from pilocarpine, we get the same results. The latency to the onset of generalized convulsive seizures (GS) was higher in sh *Lrp4* lentivirus trans-infected mice than in control mice (Fig. 5f). These results indicated that the threshold of seizures was upregulated by the reduction of Lrp4 in hippocampus.

## Discussion

Lrp4 is critical in the development and plasticity of synapses, and also in some kinds of nerve system diseases such as MG [28, 29]. Our results identified that Lrp4 also played a significant role in seizures regulation. Firstly, we found that Lrp4 expression level (both mRNA and protein) significantly decreased in the hippocampus of pilocarpine treated seizures mice (Fig. 1), and glutamate treatment reduced the Lrp4 expression level in cultured hippocampal astrocytes (Fig. 2a–c), which could be partially attenuated by NMDA receptor antagonist AP5 (Fig. 2d–f). Furthermore, we found that seizures induced by pilocarpine increased mmu-miR-351-5p expression level in vivo, and glutamate increased mmu-miR-351-5p expression level in cultured hippocampal astrocytes in vitro (Fig. 3b, c), which also could be partially attenuated by NMDA receptor antagonist AP5 (Fig. 3d). After treated hippocampal astrocytes with miR-351-5p mimics, Lrp4 expression level decreased (Fig. 4b–d). We also found that miR-351-5p inhibitors attenuated glutamate induced Lrp4 expression reduction in cultured hippocampal astrocytes (Fig. 4e–g). These results indicated that increasing glutamate during seizures induced by pilocarpine increased miR-351-5p expression in hippocampal astrocytes via activating astrocytic NMDA receptor, then decreased Lrp4 expression level in hippocampal astrocytes. At last, we stereotactic injected sh *Lrp4* lentivirus into mouse hippocampus to decrease LPR4 expression level in hippocampus (Fig. 5a–c), and found that the threshold of seizures of mice was upregulated (Fig. 5d–f). Together, these results indicated that high released glutamate during pilocarpine induced seizures decreased Lrp4 expression level in hippocampal astrocytes by increasing miR-351-5p in hippocampal astrocytes via activating astrocytic NMDA receptor, and the reduction of Lrp4 expression in hippocampus increased mice seizures threshold.

Seizure is a process result from an imbalance between excitatory and inhibitory activity within a neuronal network [30]. Many different neurobiological dysfunction contributes to this process, such as: neurogenesis [31], proinflammatory processes (interleukin 1β, TGFβ) [32, 33], neuronal voltage and ligand gated ion channels [34], neurotransmitter release or uptake [35], intracellular signaling cascades (BDNF, Trk, mTOR) [36–39], astrocytes and microglia [40]. Among them, a growing body of evidence supports that neuron-astrocytes interaction plays a very important role. Previous studies have revealed that adenosine level is rapidly upregulated as an acute response to SE, thus enhancing the protective functions of the adenosine system via increased activation of A1Rs [41]. Furthermore, the reduction of Lrp4 expression in *GFAP-Lrp4*^−/−^ mice elevated the releasement of ATP from hippocampal astrocytes, which increased the threshold of seizures via increased activation of A1Rs by adenosine (a product of ATP hydrolysis) [7, 8]. We found that seizures induced by pilocarpine increased miR-351-5p expression in hippocampal astrocytes via activating astrocytic NMDA receptor (Figs. 3, 4), then decreased Lrp4 expression level in hippocampal astrocytes (Figs. 1, 2). Locally reduction of Lrp4 expression level in hippocampus by sh *Lrp4* lentivirus trans-infection increased the threshold of seizures of mice (Fig. 5). All these data indicated that hippocampal astrocytes may potentially exert acute anti-seizures actions by increasing ATP release via downregulating Lrp4 expression level through sensing the over released glutamates by seizures. All of above findings supported that as a seizures self-regulation mechanism, astrocytic Lrp4 appears to serve as a negative feedback factor to repress the epileptic condition. It provides a new potential therapeutic target for seizures regulation.

In neuromuscular system, LPR4 is highly expressed regionally, mainly expressed in the areas with high neuronal activity (NMJ, hippocampus). High neuronal activity enhances the expression of many genes to alter structural and functional properties of the brain, such as BDNF, NRG1 [42, 43], and so on. So whether is the regional expression of Lrp4 due to high neuronal activity? On the contrary, our finding is that high neuronal activity induced by seizures rapidly depressed the expression of Lrp4, which indicated high neuronal activity is an acute inhibition signal for the expression of Lrp4. Why? It has been well studied that the mechanisms of high AChR concentration at MNJ is the result of coordination of positive and negative signals. As a negative signal, neuronal activity released ACh suppresses AChR synthesis, transport to the cell membrane, clustering or anchoring, and stability in entire muscle fibers [44, 45]. As the positive signaling, agrin produced by nerve terminals and deposited in synaptic basal lamina promotes the recycling of AChRs, increases their metabolic stability, enhances a vesicle dependent transport of AChRs to the synaptic membrane [46, 47]. So whether the Lrp4 highly concentrates at NMJ and regional expression of Lrp4 in mice brain are the result of coordination of positive and negative signals, liking AChR? We uncovered that glutamate released by high neuronal activity suppressed Lrp4 expression in hippocampal astrocytes through increasing miR-351-5p expression via activating astrocytic NMDA receptor. So it could be proposed that to forming Lrp4 regional expression, a positive signal must be existed which is worthy to further discover.

## Conclusions

In conclusion, our study demonstrated that high released glutamate during seizures induced by pilocarpine decreased the expression of Lrp4 and increased miR-351-5p in hippocampal astrocytes via activating astrocytic NMDA receptor. The reduction of Lrp4 expression was the result of increasing miR-351-5p in hippocampal astrocytes. Locally reducing Lrp4 expression in hippocampus increased mice seizures threshold. All above findings indicated that Lrp4 in hippocampal astrocytes serves as a negative feedback factor in seizures.

## Methods

### Experimental animals

Adult male C57/B6 mice were used for experiments. *Lrp4-LacZ* reporter mice were from KOMP (VG15248). In all studies, at least three pairs of mice from same litters were used. Significant efforts are also made to minimize the total number of animals used while maintaining statistically valid group numbers. Mice were group-housed in ventilated cages (no more than 5 per cage) in a temperature-controlled room with a 12-h light/dark cycle. Mice had access to water and rodent chow diet ad libitum. All experiments with animals were approved by the Institutional Animal Care and Use Committee of Nanchang University.

### Status epilepticus (SE) induction

Pilocarpine induced SE model as described previously [19]. Adult C57/B6 mice (8–12 weeks) were injected with scopolamine (2 mg/kg, i.p.) to block peripheral side effects. 30 min later, mice were injected with pilocarpine in 0.9% saline (300 mg/kg, i.p.) or 0.9% saline (same volumes). At different time points, the hippocampus was removed from pilocarpine injected mice which developed status epilepticus or 0.9% saline injected control mice for RNA and protein extraction.

### Primary astrocyte culture

Hippocampal astrocytes were cultured as described previously with modification [16]. Briefly, hippocampus was dissociated from P3 C57/B6 mice, and digested in HBSS containing 0.1% trypsin for 10–15 min. Cells were seeded on poly-l-lysine-coated 100 mm culture dishes at high density (1 × 10^7^/dish) in culture solution (DMEM medium with 10%FBS, 1 IU/ml penicillin, and 100 g/ml streptomycin). Every 3 days, after shacked extensively to remove oligodendrocytes, microglia, and neurons, medium of each dishes were changed to new culture solution. At DIV 10, 90% glia cells were GFAP-positive astrocytes. Astrocytes were trypsinized and distributed. When cell confluence quantification reached proper density, astrocytes were treated with different time intervals.

### RNA isolation, reverse transcription, and quantitative real time PCR

For RNA isolation, 1 ml Trizol LS Reagent (Invitrogen, USA) was added to mouse hippocampus tissues, primary hippocampal astrocytes. After an addition of 200 µl chloroform, RNA was precipitated with isopropyl alcohol. After washed with 75% ethanol, RNA was dissolved in RNase free water. The concentration and purity of RNA were determined at 260/280 nm using a nanodrop spectrophotometer (Thermo Fisher Scientific, Wilmington, DE, USA). Reverse transcription of mRNA was performed using a Fermentas reverse transcriptase system. Total RNA were reverse-transcribed into cDNA using oligo dT primers. PCR primers were designed using the Universal Probe Library of Roche on the basis of the reported mRNA sequences. The primers used in qRT PCR are listed as follows: *Lrp4* F: 5′-GTGTGGCAGAACCTTGACAGTC-3′, R: 5′-TACGGTCTGAGCCATCCATTCC-3′; *Gapdh* F: 5′-GAGGGCATGGGTCAGAAG-3′, R: 5′-GAGGCGTACAGGGATAGCAC-3′. Quantitative real time PCR was measured using a Thermo Scientific Maxima SYBR Green/ROX qPCR Master Mix (2×) (Cat. K0221) following the procedures laid out by the manufacturer. qPCR was run on a Roche Lightcycler 480 (Roche Applied Science, Basel, Switzerland), according to the instructions of the manufacturer. Data analysis was performed with the software provided by the manufacturer. Quantitation cycle (Cq) values were normalized to *Gapdh*, which was used as an internal control.

### Stem-loop micro-RNA RT-qPCR

micro-RNA expression was analyzed using Stem-loop micro-RNA RT-qPCR assay. Stem-loop micro-RNA RT and qPCR primers were listed in support information Table 1. cDNA was generated by reverse transcription using 1 µg RNA as template. Stem-loop micro-RNA RT primers were combined to transcribe the total RNA. qPCR was performed using the Thermo Scientific Maxima SYBR Green/ROX qPCR Master Mix (2×) (Cat. K0221) and run on a Roche Lightcycler 480 (Roche Applied Science, Basel, Switzerland), following the manufacturer's protocol and instructions. Data analysis was performed with the software provided by the manufacturer. Quantitation cycle (Cq) values were normalized to U6 small nuclear RNA, which was used as an internal control.

**Table 1:** Stem-loop RT and RT-quantitative polymerase chain reaction primer sequences

Gene	Sequence (5′ → 3′)
U6 (F)	CTCGCTTCGGCAGCACA
U6 (R)	AACGCTTCACGAATTTGCGT
mmu-miR-125a-5p (RT)	GTCGTATCCAGTGCAGGGTCCGAGGTATTCGCACTGGATACGACTCACAGGT
mmu-miR-351-5p (RT)	GTCGTATCCAGTGCAGGGTCCGAGGTATTCGCACTGGATACGACCAGGCTCA
mmu-miR-6367 (RT)	GTCGTATCCAGTGCAGGGTCCGAGGTATTCGCACTGGATACGACTCCTGAAC
mmu-miR-125b-5p (RT)	GTCGTATCCAGTGCAGGGTCCGAGGTATTCGCACTGGATACGACTCACAAGT
mmu-miR-6394 (RT)	GTCGTATCCAGTGCAGGGTCCGAGGTATTCGCACTGGATACGACAGACCTGG
mmu-miR-125a-5p (F)	CGCGTTCCCTGAGACCCTTTA
mmu-miR-351-5p (F)	CGTTCCCTGAGGAGCCCTT
mmu-miR-6367 (F)	AGCGTTCCCTGAGACCCTG
mmu-miR-125b-5p (F)	CGCGTTCCCTGAGACCCTA
mmu-miR-6394 (F)	AGCGTTCCCTGAGTGGGG
General mmu-miR (R)	ATCCAGTGCAGGGTCCGAGG

*RT* reverse transcription, *mmu-miR* mus musculus microRNA, *F* forward, *R* reverse

### In situ X-gal assay

After quickly isolated and embedded in OCT (Tissue-Tek), mice brains were cut at 40-µm interval by coronal sections and every fourth section was collected and mounted on slides. Brain sections were fixed for 4 min in ice cold X-gal fixative solution (2 mM MgCl_2_, 5 mM EGTA with 0.2% glutaraldehyde in PBS). After being washed in PBS 3 times, brain sections were stained in X-gal staining solution (1 mg/ml X-gal, 5 mM K 3Fe(CN)_6_, 5 mM K_4_Fe(CN)_6_, 0.02% NP-40, 0.01% deoxycholate, and 2 mM MgCl_2_ in PBS) at 37 °C for 8 h. And after being washed in PBS 3 more times, brain sections were counterstained with nuclear Fast Red (Vector Labs, H-3403).

### Western blotting

Hippocampus and cultured astrocytes were homogenized and prepared in RIPA Buffer containing (50 mM Tris–HCl pH 7.4, 150 mM NaCl, 2 mM EDTA, 1 mM PMSF, 50 mM sodium fluoride, 1 mM sodium vanadate, 1% sodium deoxycholate, 1% SDS and 1% protease inhibitors cocktails). Samples were resolved on SDS/PAGE, and transferred to nitrocellulose membranes. Then the membranes were incubated in TBST buffer (100 mM Tris–HCl pH 7.4, 150 mM NaCl, 0.1% Tween-20) with 5% skim milk at RT for 1 h. After being washed 3 times with TBST buffer, the membranes were incubated in primary antibody overnight at 4 °C. And being washed 3 more times with TBST buffer, the membranes were incubated with HRP-conjugated secondary antibody (Life Technology) in the same TBST buffer with 5% skim milk at RT for 1 h. Immunoreactive bands were visualized using enhanced chemiluminescence (Pierce). Band density of interested proteins was normalized in relation to loading control.

### Transfections of microRNA mimics and inhibitors

Transfections of microRNA mimics and inhibitors were conducted using Lipofectamine3000 (Invitrogen), following the procedures laid out by the manufacturer. Cells were plated in six-well plates or 100 mm culture dishes. miR-351-5p mimics (50 nM, Ribobio, Guangzhou, China); miR-351-5p inhibitors (50 nM, Ribobio, Guangzhou, China); and controls including miR-34b-5p m NC, miR-34b-5p I NC were transfected into primary astrocytes when they reached 60–70% confluence. Transfection efficiency was found to be around 80% by flow cytometry.

### Construction of the lentivirus

The optimal sequence of small interfering RNAs against mouse *Lrp4* (5′-GCAGTGTGATGGAGACAAT-3′) was cloned into the plasmid pll3.7-GFP, which encodes a human immunodeficiency virus (HIV)-derived lentiviral vector containing a multiple cloning site for the insertion of shRNA constructs to be driven by an upstream U6 promoter and a downstream cytomegalovirus promoter-GFP (marker gene) cassette flanked by loxP sites. The scrambled shRNA plasmid was constructed by a similar process (5′-GGATGACAGCGTTAGAGTA-3′). These modified plasmids were further co-transfected into HEK293T cells with lentiviral packaging plasmids to generate *Lrp4* shRNA-expressing lentivirus, or scrambled *Lrp4* shRNA-expressing lentivirus.

### Stereotactic injection

For stereotactic injection of lentivirus, the C57/B6 mice (7–8 weeks) were anesthetized and stereotaxically injected with a virus into molecules layer (ML) of the hippocampus region (0.5 ml at 0.25 ml/min) with the following coordinates (posterior = − 1.4 mm from Bregma, lateral =  ± 1.4 mm, ventral = 1.7 mm) as previously described [48]. The mice were allowed to recover for 14 days and were handled every other day to reduce the stress associated with handling at the time of behavior testing.

### Seizures behavior analysis

Two weeks after lentivirus injections, 30 min after injection of scopolamine (2 mg/kg, i.p.), mice were injected with pilocarpine (200 mg/kg, i.p.), and followed with additional injections of pilocarpine (100 mg/kg) every 30 min. Behavioral seizures were scored based on the criteria: stage 0, no seizure; stage 1, head nodding; stage 2, sporadic full-body shaking, spasms; stage 3, chronic full-body spasms; stage 4, jumping, shrieking, falling over; stage 5, violent convulsions, falling over, death. To detect seizure onset latency, pentylenetetrazol (PTZ, 50 mg/kg, i.p.) were injected into mice, then the time of the onset of generalized convulsive seizures (GS) were recorded.

### Statistical analysis

Statistical analysis was done using the GraphPad Prism version 6.01 (GraphPad Software). All data in each group were presented as mean with SD (standard deviation). Two-tailed student t-test was used to compare data from each group. *P* < 0.05 was considered to be statistically significant.

## Supplementary information

**Additional file 1: Figure S1.** Seizures increased *Bdnf* mRNA in vivo and glutamate increased *Bdnf* mRNA in vitro. A, in vivo, relative mRNA level of *Bdnf* in hippocampus increased after pilocarpine injection, which were collected at different time point (0 h, 2 h, 4 h, 12 h) after injection. B, in vitro, relative mRNA level of *Bdnf* in cultured astrocytes increased after glutamate treating, which were collected at different time point (0 h, 2 h, 4 h, 12 h) after treatment. For each experiment, three separate experiments performed in duplicate (**p < 0.01).

## Data Availability

The datasets used and/or analyzed during the current study are available from the corresponding author on reasonable request.

## Abbreviations

Lrp4: Low-density lipoprotein receptor-related protein 4
LDLR: Low-density lipoprotein receptor
BDNF: Brain-derived neurotrophic factor
LMol: Lacunosum-moleculare layer
Mol: Molecules layer
NMDA: *N*-Methyl-d-aspartate
AP5: DL-2-amino-5-phsphonovaleic acid
PTZ: Pentylenetetrazol
NMJ: Neuromuscular junction
SE: Status epilepticus
shRNA: Short hairpin ribonucleic acid
GS: Generalized convulsive seizures
