# Supplementary figures and images for: Lrp4 in hippocampal astrocytes serves as a negative feedback factor in seizures
Source: Cell Biosci. 2020 Nov 23;10:135. doi: 10.1186/s13578-020-00498-w (PMC7684739; doi:10.1186/s13578-020-00498-w)

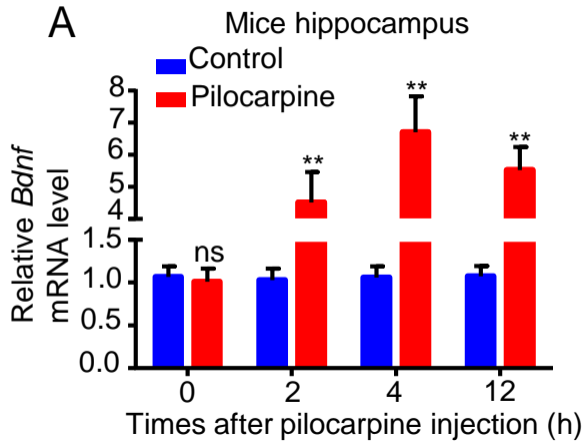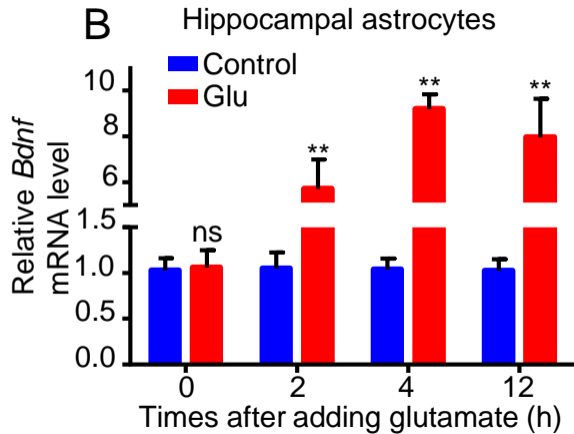

Supplement: Supplementary file 1 — Additional file 1: Figure S1. Seizures increased Bdnf mRNA in vivo and glutamate increased Bdnf mRNA in vitro. A, in vivo, relative mRNA level of Bdnf in hippocampus increased after pilocarpine injection, which were collected at different time point (0 h, 2 h, 4 h, 12 h) after injection. B, in vitro, relative mRNA level of Bdnf in cultured astrocytes increased after glutamate treating, which were collected at different time point (0 h, 2 h, 4 h, 12 h) after treatment. For each experiment, three separate experiments performed in duplicate (**p < 0.01). [file 13578_2020_498_MOESM1_ESM.pdf]
